# Efficacy of tumor necrosis factor inhibitors in hand osteoarthritis: A systematic review and meta-analysis of randomized controlled trials

**DOI:** 10.1016/j.ocarto.2023.100404

**Published:** 2023-08-12

**Authors:** Mahnuma Mahfuz Estee, Flavia M. Cicuttini, Matthew J. Page, Anita E. Wluka, Yuanyuan Wang

**Affiliations:** School of Public Health and Preventive Medicine, Monash University, Melbourne, Victoria 3004, Australia

**Keywords:** Hand osteoarthritis, Meta-analysis, Systematic review, Randomized controlled trials, Tumor necrosis factor inhibitors

## Abstract

**Objectives:**

This study aimed at systematically review the evidence for the efficacy of Tumor Necrosis Factor (TNF) inhibitors on symptoms and structural outcomes in hand osteoarthritis.

**Methods:**

Three databases were searched for randomized controlled trials examining the efficacy of TNF inhibitors in hand osteoarthritis. Two authors extracted data and assessed the risk of bias. The mean difference (MD) was calculated, and a random-effects meta-analysis was performed.

**Results:**

Four studies were identified involving 276 participants. Meta-analysis showed that TNF inhibitors had no effect on pain at 4–6 weeks (MD -0.93, 95%CI -7.41 to 5.55; 2 studies) and 24–26 weeks (MD -3.82, 95%CI -11.46 to 3.83; 2 studies) and no effect on grip strength at 12 months (MD -0.35, 95%CI -1.08 to 0.37; 2 studies). There was limited evidence for the effect of TNF inhibitors on structural outcomes at 12 months. Subgroup analysis from 2 studies showed beneficial effect of TNF inhibitors on reducing the progression of structural outcomes in hand OA patients with signs of inflammation but not in those without inflammation. The certainty of the evidence was low for the effect of TNF inhibitor on pain and moderate for the effect on grip strength.

**Conclusion:**

This study found no effect of TNF inhibitors on clinical outcomes in hand osteoarthritis over the short term (<6 weeks) and within one year, with some evidence for beneficial effect on structural outcomes.

## 1 Introduction

Hand osteoarthritis (OA) is a leading cause of chronic pain, functional impairment and stiffness in hand joints, resulting in reduced health-related quality of life [[1], [2], [3], [4]]. The age-standardised prevalence of hand OA was 44.2% in women and 37.7% in men in the general population [5]. Despite the high prevalence and disease burden, there is no recommended disease-modifying treatment proven to slow disease progression and improve outcomes in hand OA.

It is well-documented that inflammation plays an important role in the pathogenesis of hand OA [6,7]. The presence of synovitis is associated with greater pain and accelerated structural progression in hand OA [[8], [9], [10]]. Therefore, therapies targeting synovitis may offer a novel approach to managing hand OA. Tumor necrosis factor (TNF) inhibitors are a class of anti-inflammatory drugs that have been used in immunologically driven musculoskeletal conditions characterized by inflammation, including rheumatoid arthritis (RA), psoriatic arthritis and juvenile idiopathic arthritis [[11], [12], [13], [14]]. A systemic review of preclinical studies demonstrated the potential beneficial effect of TNF inhibitors for OA, evidenced by improved osteochondral viability, improved proliferation and chondrogenesis enhancing the natural repair of chondral lesions and chondroprotection by reducing inflammatory effect on cartilage [15]. Thus, TNF inhibitors have been examined as potential candidates for treating hand OA. Previous studies have shown that the progression of hand OA was reduced after treatment with TNF inhibitors in patients with RA [16] and that TNF inhibitors reduced pain and structural progression in erosive hand OA in pilot studies [17,18].

Based on data from observational studies, randomized controlled trials (RCT) have been conducted to evaluate the effect of TNF inhibitors on hand OA [[19], [20], [21], [22]]. Previous meta-analyses have examined the effect of conventional or biologic disease-modifying anti-rheumatic drugs on self-reported pain and function in patients with OA at any site [[23], [24], [25]]. In the subgroup analysis, TNF inhibitors showed no significant effect on pain improvement in hand OA [[23], [24], [25]]. However, these systematic reviews did not investigate the effect of TNF inhibitors on structural outcomes or functional outcomes (such as grip strength), nor examined the effect of TNF inhibitors on clinical outcomes based on the time points that outcomes were evaluated. Therefore, we conducted a systematic review and meta-analysis of RCTs to investigate the effect of TNF inhibitors on clinical symptoms and structural outcomes in hand OA.

## 2 Methods

The systematic review was registered on the PROSPERO (CRD42021225698) and reported in accordance with the Preferred Reporting Items for Systematic Review and meta-analysis (PRISMA) 2020 statement [26].

### 2.1 Search strategies

A systematic literature search was performed from inception to December 01, 2022 using Ovid MEDLINE(R) and Epub Ahead of Print, In-Process, In-Data-Review & Other Non-Indexed Citations, Daily and Versions(R), Ovid Embase Classic ​+ ​Embase, Ovid EBM Reviews - Cochrane Central Register of Controlled Trials using MeSH terms and key words in relation to TNF inhibitors, hand OA and RCTs (Supplementary Table 1). We also searched the reference list of included articles and published review articles.

### 2.2 Trial registers

MME searched for unpublished trials with “Completed” or “Unknown” status that met the eligibility criteria of our systematic review in US National Institutes of Health Trial Register (http://www.clinicaltrials.gov), European Clinical Trial Register (http://www.clinicaltrialsregister.eu), Australian New Zealand Clinical Trials Registry (http://www.anzctr.org.au), and International Standard Randomised Controlled Trial Number registry (http://www.isrctn.com).

### 2.3 Eligibility criteria

Studies of OA of the interphalangeal joint, carpometacarpal joint, thumb, and overall hand involvement, based on the American College of Rheumatology (ACR) criteria [27] or other valid criteria (clinical or radiological) were included. Studies with one treatment arm receiving TNF inhibitor of any generic or tradename, route, dose, duration, frequency and combination form were eligible. The comparator was placebo or any other pharmacological or non-pharmacological intervention including combined treatments for hand OA, or with TNF inhibitor at different doses, durations, and frequencies. Studies with at least one outcome related to hand OA were eligible. Studies with pain, function or grip strength as main outcomes were included. We also included studies with other outcomes, e.g. morning stiffness, tender or swollen joint count, or structural changes. RCTs, written in English and available in full-text were eligible. We excluded conference abstracts, review articles, animal studies, observational studies, non-randomized trials, and studies without a comparison group.

### 2.4 Screening and data extraction

Identified citations were exported to Covidence software. Two reviewers (MME and FMC) independently screened the title and abstract, and conducted full-text screening to identify eligible studies, with disagreements resolved by discussion. Two reviewers (MME and YW) independently extracted the data on demographics (age, sex) and number of participants, definition/description of hand OA, intervention and comparator characteristics (dose, frequency, route of administration, duration of intervention), outcome measures and time points, and results.

### 2.5 Risk of bias assessment

The risk of bias (RoB) related to pain, function, and grip strength was assessed using the Cochrane RoB 2 tool [28] by two independent reviewers (MME and YW) and reviewed by another reviewer with expertise in RoB assessment (MJP). The results were visualized using Robvis tool [29].

### 2.6 Data synthesis and reporting

We presented the summary statistics [e.g. mean and standard deviation (SD) or median with interquartile range (IQR) per group for continuous outcomes, or number and percentage of events per group for dichotomous outcomes) and effect estimates [e.g. mean difference (MD), odds ratio (OR), or relative risk (RR) with 95% confidence intervals (CI)] for all outcomes in tables (Supplementary Table 2). When necessary statistics were available, studies measuring a similar outcome domain evaluated at a similar time point regardless of type of TNF inhibitor or dose were combined in meta-analysis. We synthesized MDs for studies using the same scale to measure the outcome domain. Where necessary, IQRs were converted to SDs using the formulae presented in the Cochrane Handbook [30].

For meta-analyses, the effect estimates were synthesized using a random-effects model, assuming that clinical and methodological heterogeneity are likely to exist and have an influence on the results. All meta-analyses were conducted using the inverse-variance method, the DerSimonian and Laird method of moments estimator was used to estimate the between-study variance, and 95% CIs were calculated using the Wald type method [31]. Heterogeneity was assessed visually by inspecting the forest plots and by calculating the I^2^ statistic [32]. We did not conduct subgroup analysis due to lack of sufficient data. All statistical analyses were performed using the metan package in Stata 16 (College Station, Texas USA).

### 2.7 Assessment of risk of bias due to missing evidence and certainty in the body of evidence

We assessed RoB due to missing evidence (arising from publication bias and selective reporting bias) in the meta-analyses of pain at 24–26 weeks and grip strength at 12 months, following the framework outlined in the Cochrane Handbook [30]. We used GRADE approach to assess certainty in the body of evidence for main comparisons (i.e. TNF inhibitor vs placebo) in pain and grip strength [33]. We considered the five standard domains for downgrading evidence in GRADE to inform an overall assessment of certainty for each outcome, which was judged to be high, moderate, low and very low. MME performed the assessment, verified by MJP.

## 3 Results

### 3.1 Study selection

**Fig. 1:**
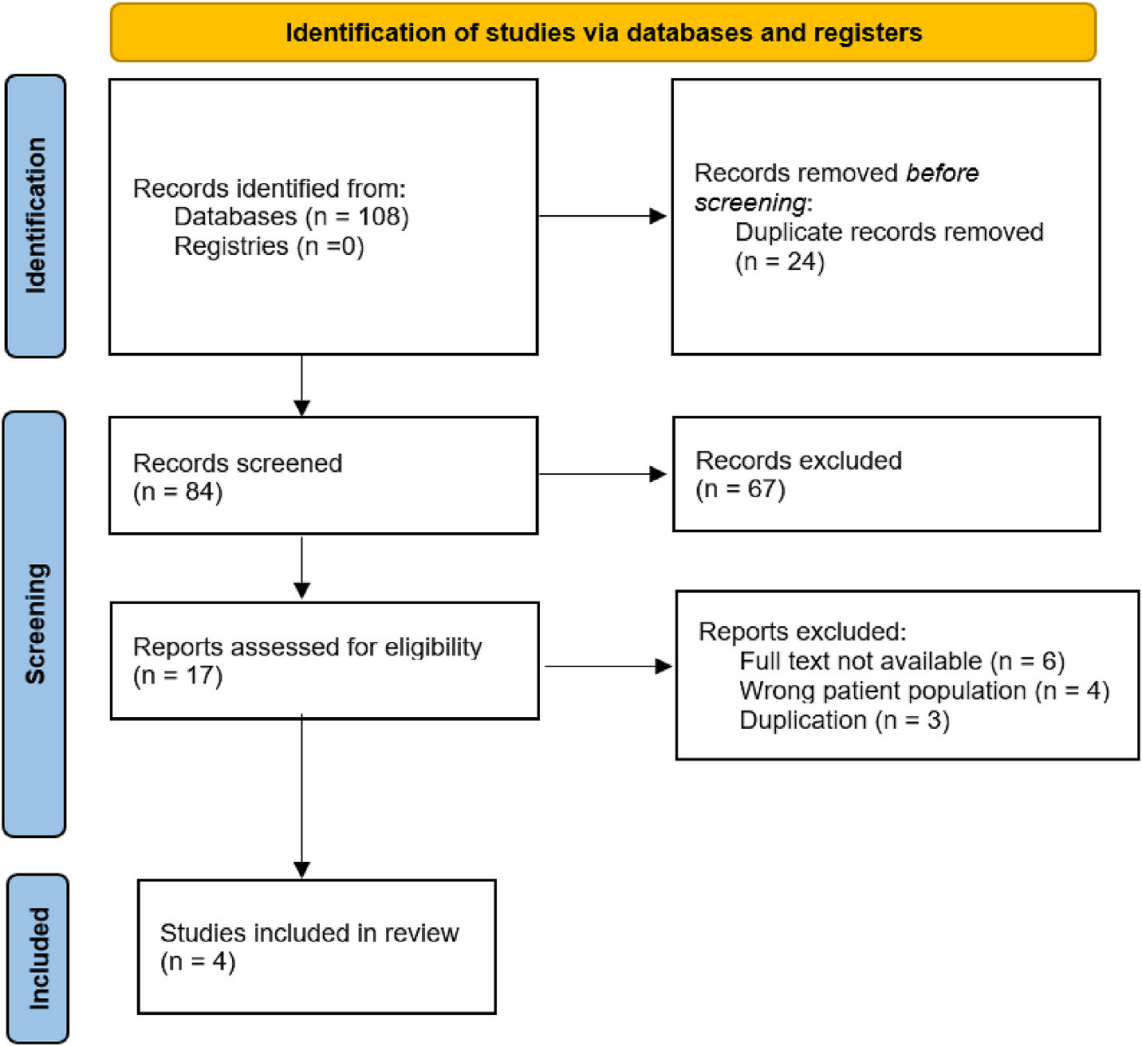
PRISMA flow diagram for efficacy of tumor necrosis factor inhibitors on hand osteoarthritis.

We identified 222 citations. After removal of duplicates, 93 citations were identified for title and abstract screening, of which 20 studies underwent full-text screening. Sixteen studies were excluded, leaving four studies eligible for inclusion (Fig. 1). No additional studies were found by searching the references of published research or review articles.

### 3.2 Trial registry search

No unpublished trials were potentially eligible for inclusion in our systematic review.

### 3.3 Overall description of included studies

Table 1 provides an overview of the four RCTs of parallel group [[19], [20], [21]] and cross-over [22] design, published between 2012 and 2018, evaluating a total of 276 participants. Two studies recruited from outpatient clinics [20,21], one study from the community, private practice and referral [22] and one study from referral [19]. The mean age ranged 59.7–62.5 years and proportion of females ranged 65.5%–85%.

**Table 1:** Characteristics of included studies.

Study Author Year Country	Study setting, design	-Avg age (years); -Number; -Female, no (%)	-Hand OA diagnosis -Additional eligible criteria -joint location	Intervention vs control	Dose frequency duration of treatment	Primary outcome	Duration of follow-up
Aitken 2018 Australia	-Community, private practice and referral -cross-over RCT	−61 years; -n ​= ​43; −33 (76.7%)	-ACR criteria, ->50 ​mm in VAS and >30 ​min morning stiffness AND ≥1 erosive joint (x-ray) with synovitis (MRI), Special Phenotype: Inflammatory OA [presence of synovitis on MRI] Erosive OA [erosive joint in x ray] -hand	Adalimumab vs placebo	40 ​mg SC injections every other week for 12 weeks, followed by 8 weeks wash out and then crossed over treatment groups for another 12 weeks	Change in VAS pain at 12 weeks	12 weeks
Kloppenburg 2018 The Netherlands	-Outpatient clinic -parallel RCT	−59.7 years; -n ​= ​90; −73 (81%)	-ACR criteria, -≥4 IP joints with osteoarthritic node or ≥1 with soft tissue swelling or erythema or >30/100 in VAS AND ≥1 IP joint with radiographic pre-erosive as defined [34]. Special Phenotype: Inflammatory OA defined by presence of soft tissue swelling/erythema or ≥1 IP joint with positive power doppler signal on ultrasound Erosive OA: radiographic erosion -IP	Etanercept vs placebo	50 ​mg weekly SC injection for 24 weeks then 25 ​mg weekly SC injection from 24 to 52 weeks	VAS pain at 24 weeks	4,8,12,24,26, 52 weeks
Chevalier 2014 France	-Recruited by referral -parallel RCT	−62.5 years; -n ​= ​83; −71 (65.5%)	-ACR criteria, -At least three IP joints symptomatic for 3 months and >40 ​mm global pain level in the last 24 ​h Special Phenotype: refractory OA (hand OA not respond to analgesics and NSAIDs) -Hand	Adalimumab vs placebo	Two 40 ​mg SC injections at a 15-day interval	Percentage of patients with ≥50% improvement in VAS pain from week 0–6	4, 6, 10, 14, 26 weeks
Verbruggen 2012 Belgium	-Out-patient rheumatology clinic -parallel RCT	−61.3 years; -n ​= ​60; −51 (85%)	-ACR criteria, -painful inflammatory episodes of the IP joint, -at least one IP joint with E (erosive) phase which is the advanced phase in the evolution if OA of the finger joint as defined [34,35]. Special Phenotype: Erosive OA: presence of erosive phase in at least one IP joint -IP	Adalimumab vs placebo	40 ​mg SC injection every 2 weeks for 52 weeks	Control of structural damage on radiography	52 weeks

ACR: American College of Rheumatology; IP: Interphalangeal; MRI: Magnetic Resonance Imaging; NSAIDs: Non-steroidal anti-inflammatory drugs; SC: Subcutaneous; RCT: Randomized controlled trials; VAS: Visual Analogue Scale.

Hand OA was diagnosed by the ACR criteria [27] in all the studies [[19], [20], [21], [22]]. Additional clinical features were used as eligible criteria for these studies. Aitken considered >50 ​mm in visual analogue scale (VAS) and >30 ​min morning stiffness [22]. Verbruggen considered painful inflammatory episodes of the interphalangeal joint [20]. Kloppenburg deemed the presence of OA node in ≥4 interphalangeal joints or soft tissue swelling in ≥1 interphalangeal joint or VAS >30 ​mm [21]. Chevalier counted ≥3 interphalangeal joints symptomatic for 3 months and >40 ​mm global pain level in the last 24 ​h [19]. Three studies included radiological evidence to define hand OA; Aitken included patients with ≥1 erosive joint on x-ray and synovitis on magnetic resonance imaging (MRI) [22], Kloppenburg [21] included patients with pre-erosive and erosive features using Verbruggen and Vey's criteria [34], and Verbruggen [20] included patients with at least one interphalangeal joint with E (erosive) phase which is the advanced phase in the evolution of OA of the finger joint as defined by Verbruggen and Vey's criteria [34,35]. All studies evaluated special phenotypes of OA: inflammatory OA [presence of synovitis on MRI [22], presence of soft tissue swelling/erythema or positive power doppler signal [21]], erosive OA [erosive joint on x-ray] [[20], [21], [22]] and refractory OA [hand OA does not respond to analgesics and NSAIDs] [19].

The duration of follow-up varied from 1 to 12 months. Two studies reported outcomes at 4–6 weeks [19,21], three studies reported at 3 months (12–14 weeks) [19,21,22], two studies reported at 6 months (24–26 weeks) [19,21], and two studies reported at 12 months [20,21].

### 3.4 Intervention

Three studies evaluated subcutaneous adalimumab [19,20,22], and one study evaluated subcutaneous etanercept [21]. Chevalier used 40 ​mg adalimumab at a 15-day interval [19]. Aitken administered injections of 40 ​mg adalimumab at every two weeks for 12 weeks in active group and placebo to the other group, and then swapped the interventions after a washout period of eight weeks [22]. Verbruggen administered 40 ​mg adalimumab fortnightly for 52 weeks [20]. Kloppenburg administered 50 ​mg etanercept weekly for the first 24 weeks and then 25 ​mg weekly for one year [21]. The control arm received a placebo (not defined) for all the studies.

### 3.5 Outcome measures

Clinical outcome measures included pain assessed using VAS and Australian/Canadian Osteoarthritis Hand Index (AUSCAN) [[19], [20], [21], [22]], function using AUSCAN and Functional Index of Hand Osteoarthritis (FIOHA) [[19], [20], [21], [22]], grip strength using a dynamometer [20,21], stiffness using AUSCAN [19,20,22], tender and swollen joint count [[19], [20], [21]]. Three studies evaluated structural outcomes [[20], [21], [22]], assessed by X-ray, ultrasonography or MRI. X-ray measures included progression of structural damage [20], prevention of erosive evaluation in presence of soft tissue swelling [20], presence of active diseases (presence of at least one new erosive joint) [20], erosive progression and signs of repair or remodelling [20,21]. Ultrasound measures included power doppler signal [21] and synovial thickening [21]. MRI measures were synovitis and bone marrow lesion [21,22].

### 3.6 Risk of bias assessment

Considering the outcomes of pain and grip strength, three studies were judged at low RoB [19,21,22], and one was judged at some concerns because of uncertainty about the randomization process [20] (Supplementary Fig. 1).

### 3.7 Effect of TNF inhibitors on pain

**Fig. 2:**
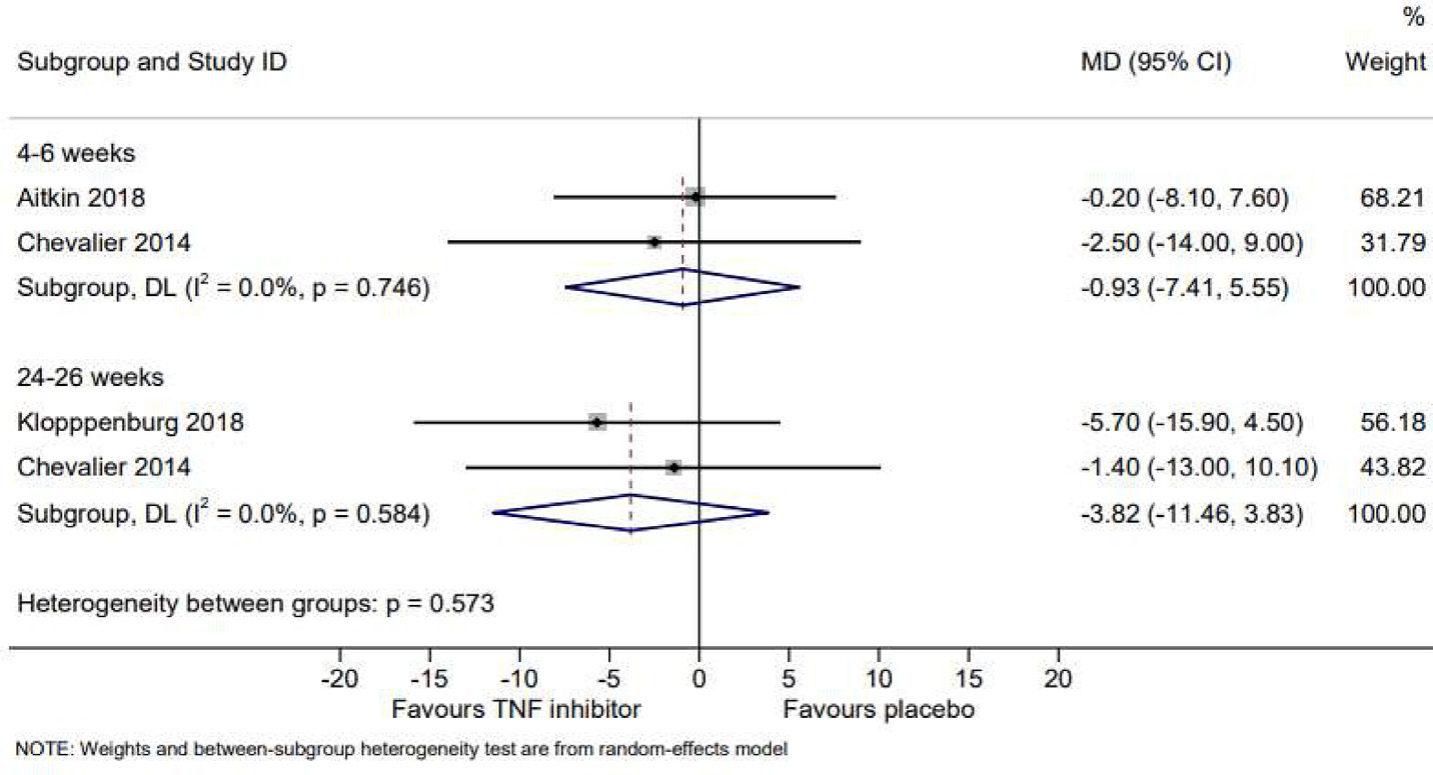
Random-effects meta-analysis of the mean difference in pain, based on tumor necrosis factor inhibitor or placebo at 4–6 weeks and 24–26 weeks.

Two studies evaluated the effect of adalimumab compared to placebo on pain at 4–6 weeks [19,22] (Table 2), with meta-analysis demonstrating no effect on reducing pain (VAS: MD -0.93, 95% CI -7.41 to 5.55) (Fig. 2). Chevalier's study showed no effect of adalimumab on significant pain improvement ≥50% at 6 weeks (RR 1.13, 95% CI 0.82 to 1.55) [19].

**Table 2:** Effect of TNF inhibitor versus control on pain and grip strength.

Study	Scale	Number TNF inhibitor	Number Control	Mean (SD) TNF inhibitor	Mean (SD) Control	Mean difference (95% CI)	Meta-analysis
**PAIN**							
**Short term (4–6 weeks)**							
Aitken 2018	VAS (0–100)	40	41	−6.1 (22.7)	−4.1 (23.0)	−0.2 (−8.1 to 7.6)	−0.93 (−7.41, 5.55)
Chevalier 2014	VAS (0–100)	38	35	−19.3 (4.2)	−16.8 (4.3)	−2.5 (−14 to 9)	
**Intermediate term (24–26 weeks)**							
Kloppenburg 2018	VAS (0–100)	45	45	39.2 (24.7)	46.5 (23.4)	−5.7 (−15.9 to 4.5)	−3.82 (−11.46 to 3.83)
Chevalier 2014	VAS (0–100)	36	33			−1.4 (−13.0 to 10.1)	
**GRIP STRENGTH**							
**Long term (12 months)**							
Kloppenburg 2018	Dynamometer	45	45			0 (−2.2, 2.1)	−0.35 (−1.08, 0.37)
Verbruggen 2012	Dynamometer	30	30	0.8 (1.2)	1.2 (1.8)		

CI: confidence interval; SD: standard deviation; TNF: tumor necrosis factor; VAS: Visual Analogue Scale.

Two studies evaluated the effect of adalimumab on pain compared to placebo at 3 months [19,22] (Supplementary Table 2). We could not perform meta-analysis due to the different design of the trials (cross-over vs parallel trial). Aitken found no better effect on pain (AUSCAN: MD -8.7, 95% CI -34.0 to 51.4; VAS: -0.7, 95% CI -9.3 to 8.0) at 12 weeks [22]. Chevalier showed reduction of pain score at 14 weeks of around 15.8 ​mm for adalimumab and around 15 ​mm for placebo [19]. Two studies evaluated the effect of TNF inhibitors (adalimumab or etanercept) on pain compared to placebo at 6 months [19,21] (Table 2), with meta-analysis showing no effect on pain (VAS: MD -3.82, 95% CI -11.46 to 3.83) (Fig. 2). Chevalier found no effect of adalimumab on 50% pain improvement at 26 weeks using VAS (RR 0.91, 95% CI 0.74 to 1.14) [19] (Supplementary Table 2).

Two studies evaluated TNF inhibitors (adalimumab or etanercept) compared to placebo on pain at 12 months [20,21] (Supplementary Table 2). Verbruggen's study showed greater change in AUSCAN-pain scale in adalimumab group compared to placebo group (5.4 vs 1.7; p ​= ​0.063) [20]. Kloppenburg et al. found no effect of etanercept on pain compared to placebo at 12 months (MD -8.5, 95% CI -18.6 to 1.6) [21].

### 3.8 Effect of TNF inhibitors on function

Two studies evaluated the effect of adalimumab on function at 4–6 weeks and showed no significant effect on functional improvement compared to placebo [19,22] (Supplementary Table 2). Chevalier used two measurement instruments and found no effect (FIHOA/Dresier: MD -0.3, 95% CI -2.7 to 2.2; Cochin hand functional index: MD -0.8, 95% CI -7.3 to 5.7) [19]. Aitken found no effect on function as assessed by AUSCAN (MD 23.2, 95% CI -34.1 to 80.5) [22].

Only Aitken et al. examined the effect of adalimumab on function and stiffness using AUSCAN subscales at 3 months, with no effect observed for function (MD 18.5, 95% CI -46.4 to 83.5) and stiffness (MD 3.3, 95% CI -5.5 to 12.1) [22] (Supplementary Table 2).

Two studies evaluated the effect of TNF inhibitors on function at 6 months [19,21] (Supplementary Table 2). Kloppenburg et al. evaluated the effect of etanercept using FIOHA and found no effect (MD 0, 95% CI -1.7 to 1.8) [21]. Chevalier et al. evaluated the effect of adalimumab using two instruments and found no effect [Dreser: MD 0, 95% CI -2.6 to 2.6; cochin hand functional index: MD 0.5, 95% CI -6.9 to 7.8] [19].

Two studies evaluated effect of TNF inhibitors on function compared to placebo at 12 months [20,21] (Supplementary Table 2). Verbruggen et al. showed no effect of adalimumab on change in AUSCAN function score compared to placebo (1.2 vs 2, p ​= ​0.133) [20]. Kloppenburg's study found no effect of etanercept on functional improvement (FIHOA: MD 0.0, 95% CI -2.4 to 2.3) [21].

**Fig. 3:**
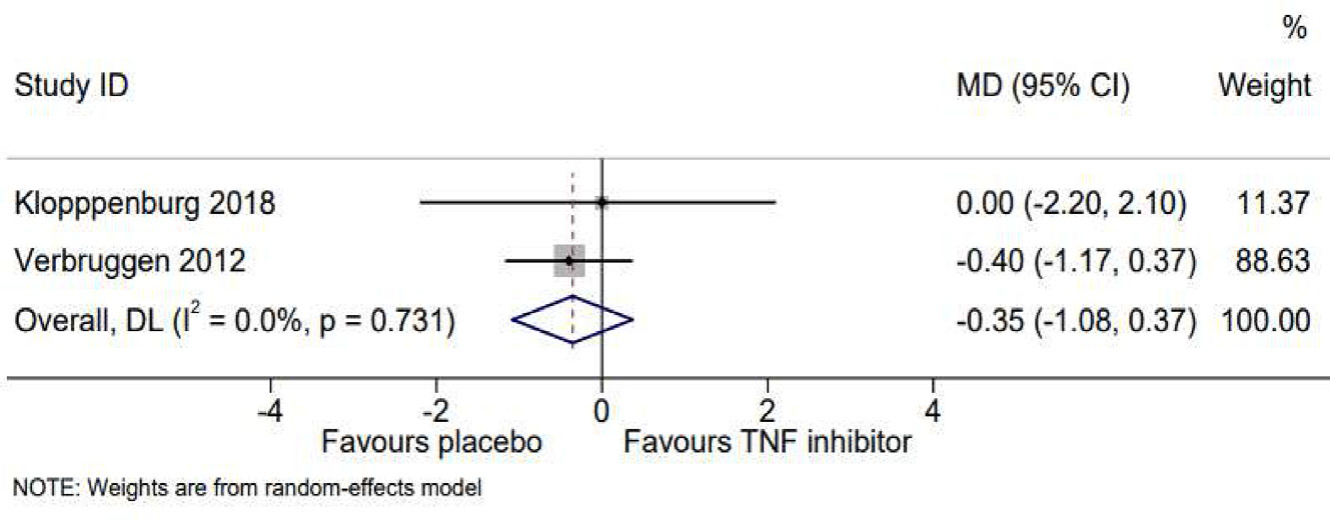
Random-effects meta-analysis of the mean difference in grip strength, based on tumor necrosis factor inhibitor or placebo at 12 months.

One study evaluated the effect of etanercept on grip strength at 6 months (24 weeks) and showed no effect (MD 0.4, 95% CI -1.7 to 2.4) [21]. Two studies examined the effect of adalimumab or etanercept on grip strength at 12 months [20,21] (Table 2), with meta-analysis showing no effect (MD -0.35, 95% CI -1.08, 0.37) (Fig. 3).

### 3.9 Effect of TNF inhibitors on structural outcome

Aitken et al. examined the radiographic and MRI outcomes at 3 months and there was no significant improvement in synovitis (RR 1.2, 95% CI 0.3 to 4.6) or bone marrow lesion score (RR 0.7, 95% CI 0.1 to 4) in the adalimumab group compared to the placebo group [22] (Supplementary Table 2).

Two studies examined the effect of TNF inhibitors (adalimumab or etanercept) on structural outcomes at 12 months [20,21] (Supplementary Table 2). Verbruggen et al. found a statistically significant effect of adalimumab on reducing erosive evolution of soft tissue swelling (OR 4.57, 95% CI 1.46 to 14.3), but no effect on development of new erosive joint (26.7% vs 40%) or progression of structural damage (OR 1.43, 95% CI 0.65 to 3.16) [20]. This study found no effect of adalimumab on erosive progression and signs of repair or remodelling at 6 and 12 months using the Ghent University Scoring System (GUSS) [36] which detected progression over a shorter period of time in erosive OA of the interphalangeal joints [20]. Kloppenburg et al. found an effect of etanercept on reducing radiographic progression using change in GUSS score (MD 2.9, 95% CI 0.5 to 5.4) and bone marrow lesion (MD -0.2, 95% CI -0.4 to −0.1), with no effect on synovitis (MD 0.03, 95% CI -0.2 to 0.3), synovial thickening (median −0.3, IQR -1.9 to 1.3), joints with power doppler (median −0.01, IQR -0.7 to 0.7), erosive progression (3.3% vs 3.5%), or remodelling (8% vs 5.2%) [21].

Two studies performed subgroup analysis based on presence or absence of signs of inflammation [20,21]. Kloppenburg et al. found that etanercept resulted in larger improvement in GUSS score and more reduction in bone marrow lesion scores compared to placebo in participants with signs of inflammation (soft tissue swelling, positive Doppler signal on ultrasound, or MRI-detected synovitis present), with no effect observed in those without signs of inflammation [21]. In the study by Verbruggen et al., patients treated with adalimumab showed significantly less change in GUSS scores towards progression and significantly reduced numbers of interphalangeal finger joints that progressed to erosive disease compared to those treated with placebo in presence of palpable effusion, with no effect seen in those without palpable effusion [20].

### 3.10 Effect of TNF inhibitors on other outcomes

Two studies showed no beneficial effect of adalimumab on the number of painful joints under digital pressure or without pressure, number of swollen joints, or morning stiffness at 4–6 weeks [19,22] (Supplementary Table 2). Adalimumab and etanercept showed no significant effect on number of painful joints under digital pressure or without pressure [19,21] or stiffness [19] at 6 months (Supplementary Table 2). Chevalier's study found significant effect of adalimumab on number of swollen joints (MD -1.9, 95% CI -3.2 to −0.6) ​[19] at 6 months, while Kloppenburg's study found no effect of etanercept on swollen joints (MD -0.03, 95% CI -0.8 to 0.8) [21]. Adalimumab and etanercept showed no significant effect on swollen or tender joint at 12 months [20,21] (Supplementary Table 2).

### 3.11 Assessment of risk of bias due to missing evidence and certainty in the body of evidence

There was some concern about RoB due to missing evidence in meta-analyses of pain (Supplementary Table 3). The RoB due to missing evidence was considered low in the meta-analysis of pain (Supplementary Table 4). The certainty in the body of evidence from our meta-analysis of pain at 24–26 weeks was moderate due to concerns about imprecision (Supplementary Table 5) and was low for the meta-analysis of grip strength at 12 months due to concerns about study risk of bias and imprecision (Supplementary Table 6).

## 4 Discussion

Our systematic review and meta-analysis demonstrated that TNF inhibitors (Adalimumab and Etanercept) had no effect on improving pain or function at any time point within 12 months. These TNF inhibitors did not improve grip strength over 6–12 months. These results indicate that TNF inhibitors (Adalimumab and Etanercept) showed no significant effect on any clinical outcomes within 12 months. There was no clear evidence for an effect of these TNF inhibitors on inflammation biomarkers on imaging. However, there was evidence from a single study that adalimumab reduced erosive evolution of soft tissue swelling and that etanercept reduced radiographic progression and bone marrow lesions at 12 months, suggesting a potential effect of TNF inhibitors on slowing disease progression of hand OA.

Our meta-analysis of two studies showed no benefit of TNF inhibitors for improving pain at 4–6 weeks or 24–26 weeks (two studies with low RoB) and grip strength at 12 months (one with low RoB and one with some concerns) in hand OA. We also found no effect of TNF inhibitors on functional improvement in hand OA. Our findings are consistent with the previous meta-analyses examining the effect of biologic agents for the treatment of OA at any site, with subgroup analysis showing no significant effect of TNF inhibitors on pain relief in hand OA [23,24].

Three studies evaluated the effect of TNF inhibitors on joint structures [[20], [21], [22]]. Although two studies showed no effect of TNF inhibitors on synovitis [21,22], two studies demonstrated some evidence for an effect of TNF inhibitors on slowing disease progression, evidenced by reducing radiographic progression, bone marrow lesions, and erosive evolution of soft tissue swelling [20,21]. Approximately 50% of those with symptomatic hand OA have evidence of synovitis [37,38] which is associated with pain and disease progression [37,39]. In a rat model with OA, TNF inhibitor reduced the expression of inflammatory cytokines in synovial fibroblasts [40]. TNF inhibitors have effects on modifying structure in the OA joints with the presence of inflammation [15,41]. Although our results did not show a clear evidence for TNF inhibitors in reducing inflammation on imaging, there is some evidence that TNF inhibitors might have an effect on slowing disease progression. Two studies showed beneficial effect of TNF inhibitors on reducing the progression of structural outcomes compared to placebo in hand OA patients with signs of inflammation but not in those without inflammation [20,21]. These results suggest that hand OA patients with the inflammation phenotype are most likely to benefit from TNF inhibitors, similar to the findings of knee OA where TNF inhibitor was found to be a successful treatment for inflammatory knee OA [42]. This suggests that the pathological mechanisms driving hand OA are complex and that although inflammation has a role [43], targeting only one inflammatory pathway may not be enough to improve clinical and disease outcomes. It may also be that better phenotyping of individuals with hand OA may identify subgroups most likely to benefit from therapies such as TNF inhibitors or other targeted therapies.

There are several strengths of our systematic review. To our knowledge, it is the first systematic review and meta-analysis evaluating the efficacy of TNF inhibitors on symptoms and structural outcomes in hand OA. It was also reported in accordance with the PRISMA 2020 guideline. We performed a comprehensive search in three databases in addition to clinical trial registries to identify unpublished trials. The RoB 2 tool and ROB-ME tool were used to assess risk of bias and the risk of bias due to missing evidence. However, our systematic review and meta-analysis have some limitations. There was heterogeneity across studies in terms of study populations, formulation and dosage of TNF inhibitors, protocol and duration of treatment, outcome measures, and length of follow-up. This clinical heterogeneity, along with lack of available data, precluded the meta-analysis for most of the outcomes. Additionally. the results of this systematic review and meta-analysis are limited to Adalimumab and Etanercept. The GRADE assessment showed the certainty of the evidence for the efficacy of TNF inhibitors was moderate for pain and low for grip strength. With limited number of studies and moderate sample size of included studies, our systematic review did not show clear evidence for an effect of TNF inhibitors on clinical or structural outcomes.

Our systematic review and meta-analysis found no significant effect of TNF inhibitors on clinical outcomes within one year. There was some evidence for a potential effect of TNF inhibitors on slowing disease progression. There is a growing interest in treating hand OA with inflammation, and our systematic review of four RCTs with moderate sample size and significant heterogeneity provides some evidence for a potential effect of TNF inhibitors on reducing structural progression in hand OA with signs of inflammation, which warrants further investigation. In the absence of any effective disease-modifying therapy in hand OA, TNF inhibitors may have utility in carefully selected patients with evidence of inflammatory changes in hand OA. Our study highlights the need for further studies examining the effect of TNF inhibitors in hand OA with the inflammation phenotype, with adequate sample size and duration of follow-up, so that any effect on symptoms and structural outcomes can be adequately determined.

## Availability of data and materials

The datasets generated and/or analysed during the current study are available in the Open Science Framework, https://osf.io/8kud4/?view_only=9db1ef6102be4df5942c5f8dbba3de0e.

## Funding

MME is a recipient of Bangabandhu Science and technology Fellowship from Ministry of Science and Technology, Government of the People's Republic of Bangladesh for her PhD. FMC is the recipient of 10.13039/501100000925National Health and Medical Research Council Investigator Grant (APP1194829). MJP is supported by an Australian Research Council Discovery Early Career Researcher Award (DE200101618). AEW is the recipient of the Fellows Career Development Fellowship from the Royal Australasian College of Physicians. YW is the recipient of NHMRC Translating Research into Practice Fellowship (APP1168185). The funding bodies had no role in the study design and conduct of the study; collection, analysis and interpretation of the data; preparation, review, or approval of the manuscript; and decision to submit the manuscript for publication.

## Authors' contributions

MME: title and abstract screening, full text screening, data extraction, risk of bias assessment, GRADE assessment, data analysis, interpretation of results, drafting manuscript, approval of the final manuscript. FMC: conceptualization, systematic review and meta-analysis planning, title and abstract screening, full text screening, interpretation of results, critical revision of the manuscript for important intellectual content, and approval of the final manuscript. MJP: risk of bias assessment, meta-analysis, GRADE assessment, interpretation of results, critical revision of the manuscript for important intellectual content, and approval of the final manuscript. AEW: interpretation of results, critical revision of the manuscript for important intellectual content, and approval of the final manuscript. YW: conceptualization, systematic review and meta-analysis planning, data extraction, risk of bias assessment, interpretation of results, drafting manuscript, critical revision of the manuscript for important intellectual content, and approval of the final manuscript.

## Declaration of competing interest

None.
