# Supplementary material for: Efficacy of tumor necrosis factor inhibitors in hand osteoarthritis: A systematic review and meta-analysis of randomized controlled trials
Source: Osteoarthr Cartil Open. 2023 Aug 12;5(4):100404. doi: 10.1016/j.ocarto.2023.100404 (PMC10462838; doi:10.1016/j.ocarto.2023.100404)

***Supplementary Table 1: Search strategy for systematic review in Ovid MEDLINE(R) and Epub Ahead of Print, In-Process, In-Data-Review & Other Non-Indexed Citations, Daily and Versions(R)/Embase/Cochrane, Ovid Embase Classic+Embase, Ovid EBM Reviews - Cochrane Central Register of Controlled Trials:***

|  | Search term/ key words |
| --- | --- |
| 1 | (hand* or "hand joint*" or "ïntermetacarpal joint*" or finger* or "finger joint*" or "carpal joint*" or digit* or "carpometacarpal joint*" or "metacarpophalangeal joint" or thumb* or metacarpus or "Thumb Carpometacarpal Joint" or "first metacarpal-carpal" or carpometacarpal or interphalangeal or "distal interphalangeal" or "proximal interphalangeal" or intermetacarp* or interphalang* or intercarp* or carpometacarp* or metacarpophalang* or metacarp* or CMC or IP or "thumb base*" or TB).mp. |
| 2 | (Osteoarthritis or Osteo-arthritis or osteoarthr* or osteo-arthr* or Osteo-arthritis or osteoarthr* or osteo-arthr* or osteoarthrosis or "degenerative arthritis" or "erosive osteoarthritis" or "arthrosis" or "arthroses" or "OA" or HOA).mp. |
| 3 | ("randomized controlled trial" or "randomised controlled trial" or "controlled trial" or "multicentre randomised controlled trial" or "multicentre randomized controlled trial" or "randomized single-blind controlled trial" or "randomised single-blind controlled trial" or "Prospective, Randomized Study" or "Prospective, Randomised Study" or "double-blind, randomised, placebo-controlled trial" or "double-blind, randomized, placebo-controlled trial" or "randomised study" or "randomized study" or "Randomized Trial" or "Randomised Trial" or "randomized, single-blind, prospective study" or "randomised, single-blind, prospective study" or "randomised, double-blind, placebo-controlled trial" or "randomized, double-blind, placebo-controlled trial" or "randomized clinical trial" or "randomised clinical trial" or "clinical trial" or "randomised double-blind placebo-controlled crossover trial" or "randomized double-blind placebo-controlled crossover trial" or "randomised double-blind placebo-controlled trial" or "randomized double-blind placebo-controlled trial").mp. |
| 4 | (infliximab or remicade or infliximab or 170277-31-3 or etanercept or enbrel or etanercept or 185243-69-0 or adalimumab or humira or adalimumab or 331731-18-1 or golimumab or simponi or golimumab or 476181-74-5 or anti-tnf therapy or anti-tnf alpha or anti tumor necrosis factor alpha therap* or tumor necrosis factor antagonist* or TNF blocker* or tumor necrosis factor blocker* or tumor necrosis factor inhibit* or TNFR Fc fusion protein* or Infliximab* or Adalimumab* or AntiTNF* or Certolizumab).mp. |
| 5 | 1 and 2 and 3 and 4 |
| 6 | Limit to English language |

***Supplementary Table 2: Description and results of the included studies***

| **Study**  **Year**  **country** | **outcome** | **Outcome measures** | **End points** | **intervention** | **control** | **Mean difference (95% CI)/OR (95% CI)/RR (95% CI)/Median (IQR)** | **P value** |
| --- | --- | --- | --- | --- | --- | --- | --- |
| **Subcutaneous** | | | | | | | |
| **Adalimumab vs placebo** | | | | | | | |
| Verbruggen (2012), Belgium |  |  |  |  |  |  |  |
|  | Progression of structural damage (control vs intervention) | Evolution from N, S, or J to E phase on radiograph defined by Verbruggen and Veys | 12  months | 9/419 (2.1%) | 15/429 (3.6%) | OR 1.43 (95% CI 0.65 to 3.16) | 0.37 |
|  | Erosive evolution in presence of soft tissue swelling (subgroup analysis) | Evolution from N, S, or J to E phase on radiograph defined by Verbruggen and Veys | 12 months | 3/81 (3.7%) | 9/62 (14.5%) | OR 4.57 (95% CI 1.46 to 14.3) | 0.009 |
|  | Active disease | Presence of at least one new erosive joint | 12  months | 8/30 (26.7%) | 12/30 (40.0%) |  | 0.09 |
|  | Erosive progression and signs of repair or remodelling | GUSS | Difference between baseline and 6 months  Difference between baseline and 12 months |  |  | Joints with palpable swelling: mean difference −20.0  (SE 9.9)  Non-swollen interphalangeal joints: No significant change in GUSS score (figure 3b)  Joints with palpable swelling: higher GUSS scores in adalimumab group vs placebo  Non-swollen interphalangeal joints: No significant change in GUSS score (figure 3b) |  |
|  | Pain (data shown as mean diff (SD) in change of score from baseline to week 52 | AUSCAN | Baseline  12  months | 20.4 (9)  5.4 (9.8) | 25.1 (11.1)  1.7 (13.1) |  | 0.063 |
|  | Stiffness (data shown as mean diff (SD) in change of score from baseline to week 52 | AUSCAN | Baseline  12  months | 5.1 (2.3)  0.4 (2.1) | 5.5 (2.6)  0.1 (3.0) |  | 0.721 |
|  | Function (data shown as mean diff (SD) in change of score from baseline to week 52 | AUSCAN | Baseline  12  months | 48.4 (20.2)  1.2 (18.5) | 54.1 (21.1)  2.0 (17.9) |  | 0.133 |
|  | No of tender joints (16 joints were assessed) |  | Baseline  12  months | 3.3 (2.7)  0.7 (2.3) | 5.5 (3.7)  1.4 (4.8) |  | 0.238 |
|  | No of joints with palpable effusion (16 joints were assessed) |  | Baseline  12  months | 3.2 (3)  1.7 (2.7) | 2.5 (2.5)  1.0 (2.4) |  | 0.814 |
|  | Maxiimum grip strength on dominant hand  (data shown as mean diff (SD) in change of score from baseline to week 52 |  | Baseline  12  months | 18.4 (9.6)  0.8 (1.2) | 19.4 (10.1)  1.2 (1.8) |  | 0.398  0.231 |
|  | Maximum grip strength on non-dominant hand (data shown as mean diff (SD) in change of score from baseline to week 52 |  | Baseline  12  months | 17.2 (7.4)  0.9 (1.3) | 18.3 (9.4)  0.2 (1.1) |  | 0.258  0.281 |
| **Adalimumab vs placebo** | | | | | | | |
| Chevalier (2014), France | Pain improve≥50%  (exclusion of missing data) | VAS | 6 weeks  26 weeks | 13 (31.7%)  6 (17.1%) | 9 (27.3%)  8 (24.2%) | Mean difference 1.13 (95% CI 0.82 to 1.55)  0.91 (0.74 to 1.14) | 0.47  0.41 |
|  | Pain change from baseline | VAS | 0week  2 weeks  4 weeks  6 weeks  10 weeks  14 weeks  26 weeks | 0  -12^^  -18^^  −19.3 (4.2)  -16^^  -15.8^^  −12.4 (4.2) | 0  -7^^  -18^^  −16.8 (4.3)  -18^^  -15^^  −11.0 (4.3) | -  −2.5 (−14.0 to 9.0)  −1.4 (−13.0 to 10.1) | -  0.67  0.80 |
|  | Number of painful IP joints vs baseline | Without pressure | 6 weeks  26 weeks | −4.1 (1.0)  −4.2 (1.1) | −3.7 (1.0)  −2.7 (1.1) | −0.5 (−3.3 to 2.4)  −1.4 (−4.5 to 1.6) | 0.75  0.35 |
|  | Number of painful IP joints vs baseline | Under digital pressure | 6 weeks  26 weeks | −4.1 (1.0)  −3.6 (1.0) | −3.7 (1.0)  −2.7 (1.1) | −0.4 (−3.1 to 2.4)  −0.9 (3.7 to 2.0) | 0.80  0.55 |
|  | Number of swollen joints vs baseline |  | 6 weeks  26 weeks | −2.3 (0.4)  −3.0 (0.5) | −1.6 (0.5)  −1.1 (0.5) | 0.7 (−1.9 to 0.5)  −1.9 (−3.2 to −0.6) | 0.27  0.0062 |
|  | Morning stiffness duration vs baseline | minutes | 6 weeks  26 weeks | −13.1 (6.4)  −13.5 (7.1) | −16.1 (6.5)  −7.8 (7.4) | 3.0 (−14.4 to 20.5)  −5.8 (−25.6 to 14.1) | 0.73  0.56 |
|  | Hand Function vs baseline | FIHOA/dreiser index | 6 weeks  26 weeks | −2.2 (0.9)  −1.3 (1.0) | −1.9 (0.9)  −1.3 (1.0) | −0.3 (−2.7 to 2.2)  0.0 (−2.6 to 2.6) | 0.82  1.00 |
|  |  | Cochin Hand Functional index | 6 weeks  26 weeks | −4.0 (2.3)  −0.7 (2.6) | −3.2 (2.4)  −1.1 (2.7) | −0.8 (−7.3 to 5.7)  0.5 (−6.9 to 7.8) | 0.82  0.90 |
| **Adalimumab vs placebo** | | | | | | | |
| Aitken (2018), Australia (4)# | Change of mean pain from baseline | VAS | 4 weeks  8 weeks  12 weeks | -6.1 (22.7)  -7.7 (21.5)  -3.2 (16.7) | -4.1 (23.0)  -5.2 (27.4)  -0.8 (29.6) | -0.2 (-8.1 to 7.6)^  -0.4 (-10.2 to 9.4)^  -0.7 (-9.3 to 8.0)^ | 0.95  0.94  0.87 |
|  |  | AUSCAN-pain | 4 weeks  8 weeks  12 weeks | -31.0 (102.4)  - 41.5 (97.2)  - 20.9 (83.7) | - 12.9 (132.2)  - 18.7 (140.9)  - 4.9 (142.6) | -1.9 (-40.7 to 36.9)^  -4.3 (-52.7 to 44.1)^  -8.7 (-34.0 to 51.4)^ | 0.92  0.86  0.68 |
|  | Change of mean function from baseline | AUSCAN-function | 4 weeks  8 weeks  12 weeks | - 21.9 (166.7)  - 61.4 (189.0)  - 23.0 (136.9) | - 23.0 (169.5)  - 8.0 (198.7)  - 6.7 (215.8) | 23.2 (-34.1 to 80.5)^  -19.4 (-103.1 to 64.4)^  18.5 (-46.4 to 83.5)^ | 0.42  0.64  0.57 |
|  | Change of mean stiffness from baseline | AUSCAN-stiffness | 4 weeks  8 weeks  12 weeks | - 4.1 (23.2)  - 4.3 (21.4)  - 2.9 (22.4) | - 4.9 (25.7)  - 7.5 (25.9)  - 5.3 (28.8) | 1.0 (-8.7 to 10.7)^  4.9 (-6.6 to 16.3)^  3.3 (-5.5 to 12.1)^ | 0.84  0.39  0.45 |
|  | Improvement in synovitis score from baseline (Intervention vs placebo) | MRI: OMERACT HOAMRIS or TOMS | 12 weeks | 5/42 (12%) | 4/41 (10%) | RR 1.2 (95% CI 0.3-4.6)^ | 0.74 |
|  | Improvement in BML score from baseline | MRI: OMERACT HOAMRIS or TOMS | 12 weeks | 2/42 (5%) | 3/41 (7%) | RR 0.7 (95% CI 0.1-4)^ | 0.65 |
| **etanercept (ETA) vs placebo** | | | | | | | |
| Kloppenburg (2018), The Netherlands (7) | Pain | VAS | 24 weeks  1 year | 39.2 (24.7)  35.7 (25.1) | 46.5 (23.4)  45.4 (25.7) | Mean difference −5.7 (95% CI −15.9 to 4.5)  −8.5 (−18.6 to 1.6) | 0.27  0.10 |
|  | Function | FIOHA | 24 weeks  1 year |  |  | 1. (−1.7 to 1.8)   0.0 (−2.4 to 2.3) | 0.97  0.98 |
|  | Grip strength | dynamometer or vigorimeter | 24 weeks  1 year |  |  | 0.4 (−1.7 to 2.4)  0.0 (−2.2 to 2.1) | 0.74  0.97 |
|  | Tender joint count |  | 24 weeks  1 year |  |  | −0.4 (−1.8 to 1.0)  0.4 (−1.4 to 2.1) | 0.58  0.66 |
|  | Soft swollen joint count |  | 24 weeks  1 year |  |  | −0.03 (−0.8 to 0.8)  −0.01 (−0.9 to 0.9) | 0.94  0.99 |
|  | Joints with power doppler | USG | 24 weeks  1 year |  |  | Median −0.3 (IQR −1.04 to 0.4)  −0.01 (−0.7 to 0.7) | 0.39  0.98 |
|  | Joints with synovial thickening | USG | 24 weeks  1 year |  |  | Median 0.2 (IQR −1.02 to 1.3)  −0.3 (−1.9 to 1.3) | 0.07  0.73 |
|  | Radiographic progression | Erosive progression defined by Verbruggen-Veys scores  Remodelling defined by Verbruggen-Veys scores | 1 year  1 year | 9 (3.3%)  24 (8.0%) | 14 (3.5%)  22 (5.2%) |  | 0.86  0.13 |
|  | Radiographic progression | Change in GUSS score | 1 year |  |  | Mean difference 2.9 (95% CI 0.5 to 5.4) | 0.02 |
|  | Synovitis | MRI: synovitis score | 1 year |  |  | 0.03 (−0.2 to 0.3) | 0.81 |
|  | Bone Marrow lesion | MRI: BML score | 1 year |  |  | −0.2 (−0.4 to −0.1) | 0.001 |

OR: odds ratio; RR: relative risk; CI: confidence interval; IQR: interquartile range, SD: Standard deviation, SE: Standard Error; GUSS: Ghent University Scoring System AUSCAN: AUStralian CANadian Osteoarthritis Hand Index VAS: Visual Analogue Scale; FIHOA: Functional Index of Hand Osteoarthritis, IP: Interphalangeal join , BML: Bone Marrow Lesion, USG: Ultra sonography, MRI: Magnetic resonance imaging

*** N(%) # cross over trial, ^ adjusted for within-subject baseline difference and the order in which the participant received their treatment (eg active then placebo or placebo then active)^^ collected from graph

**Supplementary Table 3: Assess risk of bias due to missing evidence in a synthesis (complete for each synthesis)**

Responses underlined in green are potential markers for low risk of bias, and responses in red are potential markers for a risk of bias.

| **Details of the synthesis being assessed for risk of bias** | | |
| --- | --- | --- |
| **Specify the synthesis** | Random effect meta-analysis of the effect of TNF inhibitor vs placebo on pain at 24-26 weeks | |
| **Specify the synthesized result (e.g. estimate and 95% CI)** | -3.82 (-11.46, 3.83) | |
| **Specify the number of included studies and participants** | 2; 148 | |
| **Risk of bias assessment** | | |
| **Signalling questions** | **Comments** | **Response options** |
| ***The following questions relate to the within-study assessment of non-reporting bias (‘known unknowns’)*** | | |
| **4.1. Of the studies identified, was there any for which no result was available for inclusion in the synthesis, likely because of the P value, magnitude or direction of the result generated (refer to Step 2)?** | N | Y / N |
| **4.2. If Y to 4.1: Is it likely that there would be a notable change to the synthesized effect estimate if the omitted results had been included?** | NA | NA / Y / PY / PN / N / NI |
| **4.3. Of the studies identified, was there any for which it was unclear whether an eligible result was generated (refer to Step 2)?** | N | Y / N |
| **4.4. If Y to 4.3: Is it likely that there would be a notable change to the synthesized effect estimate if the potentially omitted results had been included?** | NA | NA / Y / PY / PN / N / NI |
| ***The following questions relate to the across-study assessment of non-reporting bias (‘unknown unknowns’)*** | | |
| **4.5 Do circumstances indicate potential for some eligible studies not being identified because of the P value, magnitude or direction of the results generated (refer to Step 3)?** | Y | Y / PY / PN / N |
| **4.6. If Y/PY to 4.5: Is it likely that studies not identified had results that were eligible for inclusion in the synthesis?** | PY | NA / Y / PY / PN / N |
| **4.7. If Y to 4.1 or 4.3 or Y/PY to 4.5: Does the pattern of observed study results suggest that the synthesis is likely to be missing results that were systematically different (in terms of P value, magnitude or direction) from those observed?** | PN (as they all do not have statistically significant results) | NA / Y / PY / PN / N |
| **4.8. If Y/PY/NI to 4.2, 4.4, 4.6 or 4.7: Did sensitivity analyses suggest that the synthesized result was biased due to missing results?** | N | NA / Y / PY / PN / N |
| **Risk of bias judgement** | Some concern | Low / High / Some concerns |
| Optional: What is the predicted direction of bias for this synthesis? |  | NA / Favours experimental / Favours comparator / Towards null /Away from null / Unpredictable |

Y: ‘Yes’; PY: ‘Probably yes’; PN: ‘Probably no’; N: ‘No’; NI: ‘No information’; NA: ‘Not applicable’.

**Supplementary Table 4. Assess risk of bias due to missing evidence in a synthesis (complete for each synthesis)**

Responses underlined in green are potential markers for low risk of bias, and responses in red are potential markers for a risk of bias.

| **Details of the synthesis being assessed for risk of bias** | | |
| --- | --- | --- |
| **Specify the synthesis** | Random effect meta-analysis of the effect of tnf inhibitor vs placebo on pain at 12 months | |
| **Specify the synthesized result (e.g. estimate and 95% CI)** | -0.35 (-1.08,0.37) | |
| **Specify the number of included studies and participants** | 2; 139 | |
| **Risk of bias assessment** | | |
| **Signalling questions** | **Comments** | **Response options** |
| ***The following questions relate to the within-study assessment of non-reporting bias (‘known unknowns’)*** | | |
| **4.1. Of the studies identified, was there any for which no result was available for inclusion in the synthesis, likely because of the P value, magnitude or direction of the result generated (refer to Step 2)?** | N | Y / N |
| **4.2. If Y to 4.1: Is it likely that there would be a notable change to the synthesized effect estimate if the omitted results had been included?** | NA | NA / Y / PY / PN / N / NI |
| **4.3. Of the studies identified, was there any for which it was unclear whether an eligible result was generated (refer to Step 2)?** | N | Y / N |
| **4.4. If Y to 4.3: Is it likely that there would be a notable change to the synthesized effect estimate if the potentially omitted results had been included?** | NA | NA / Y / PY / PN / N / NI |
| ***The following questions relate to the across-study assessment of non-reporting bias (‘unknown unknowns’)*** | | |
| **4.5 Do circumstances indicate potential for some eligible studies not being identified because of the P value, magnitude or direction of the results generated (refer to Step 3)?** | Y | Y / PY / PN / N |
| **4.6. If Y/PY to 4.5: Is it likely that studies not identified had results that were eligible for inclusion in the synthesis?** | PN | NA / Y / PY / PN / N |
| **4.7. If Y to 4.1 or 4.3 or Y/PY to 4.5: Does the pattern of observed study results suggest that the synthesis is likely to be missing results that were systematically different (in terms of P value, magnitude or direction) from those observed?** | PN | NA / Y / PY / PN / N |
| **4.8. If Y/PY/NI to 4.2, 4.4, 4.6 or 4.7: Did sensitivity analyses suggest that the synthesized result was biased due to missing results?** | N | NA / Y / PY / PN / N |
| **Risk of bias judgement** | low | Low / High / Some concerns |
| Optional: What is the predicted direction of bias for this synthesis? |  | NA / Favours experimental / Favours comparator / Towards null /Away from null / Unpredictable |

Y: ‘Yes’; PY: ‘Probably yes’; PN: ‘Probably no’; N: ‘No’; NI: ‘No information’; NA: ‘Not applicable’.

***Supplementary Table 5: Random effect meta-analysis of the effect of TNF inhibitor vs placebo on pain at 24-26 weeks***

|  | Risk of bias | indirectness | Inconsistency | Imprecision | Reporting bias | score | Grade rating |
| --- | --- | --- | --- | --- | --- | --- | --- |
|  | Are there limitations with the study methods? | Do the resutls not really apply to my question? | Are the results inconsistent across studie? | Are there too few people or events? | Are we missing studies or have selective studies? | Total 4 |  |
|  | None (0) | None (0) | None (0) | Serious (-1) | None (0) | 4-0-0-0-1-0=3 | moderate |
|  | Both studies with low risk | -similar to our question  -similar to our inclusion criteria | I2 =0% | (too few participants 148 in total and CI same side ????) | -is there high probability of missing results  -yes (small studies with positive result)  -comprehensive search  Yes  registry search  yes (no unpublished trials)  -less than 10 paper so could not do funnel plot  Contacted expert>? No  -all of them are in one side. Funnel plot will look like asymmetry |  |  |

***Supplementary Table 6: Random effect meta-analysis of the effect of TNF inhibitor vs placebo on grip strength at 12 months***

|  | Risk of bias | indirectness | Inconsistency | Imprecision | Reporting bias | score | Grade rating |
| --- | --- | --- | --- | --- | --- | --- | --- |
|  | Are there limitations with the study methods? | Do the resutls not really apply to my question? | Are the results inconsistent across studie? | Are there too few people or events? | Are we missing studies or have selective studies? |  |  |
|  | Serious (-1) | none | None (0) | serious (-1) | None (0) | 4-1-0-0-1-0= 2 | Low |
|  | One with some concern | -similar to our question  -similar to our inclusion criteria | I2 = 0% | (too few participants 139 in total and CI same side ?????) and not between harm and benefit | -is there high probability of missing results  -yes (small studies with positive result)  -comprehensive search  Yes  registry search  yes (no unpublished trials)  -less than 10 paper so could not do funnel plot  Contacted expert>? No |  |  |

***Supplementary Figure 1: Risk of bias assessment using RoB 2 tool considering patient reported pain***


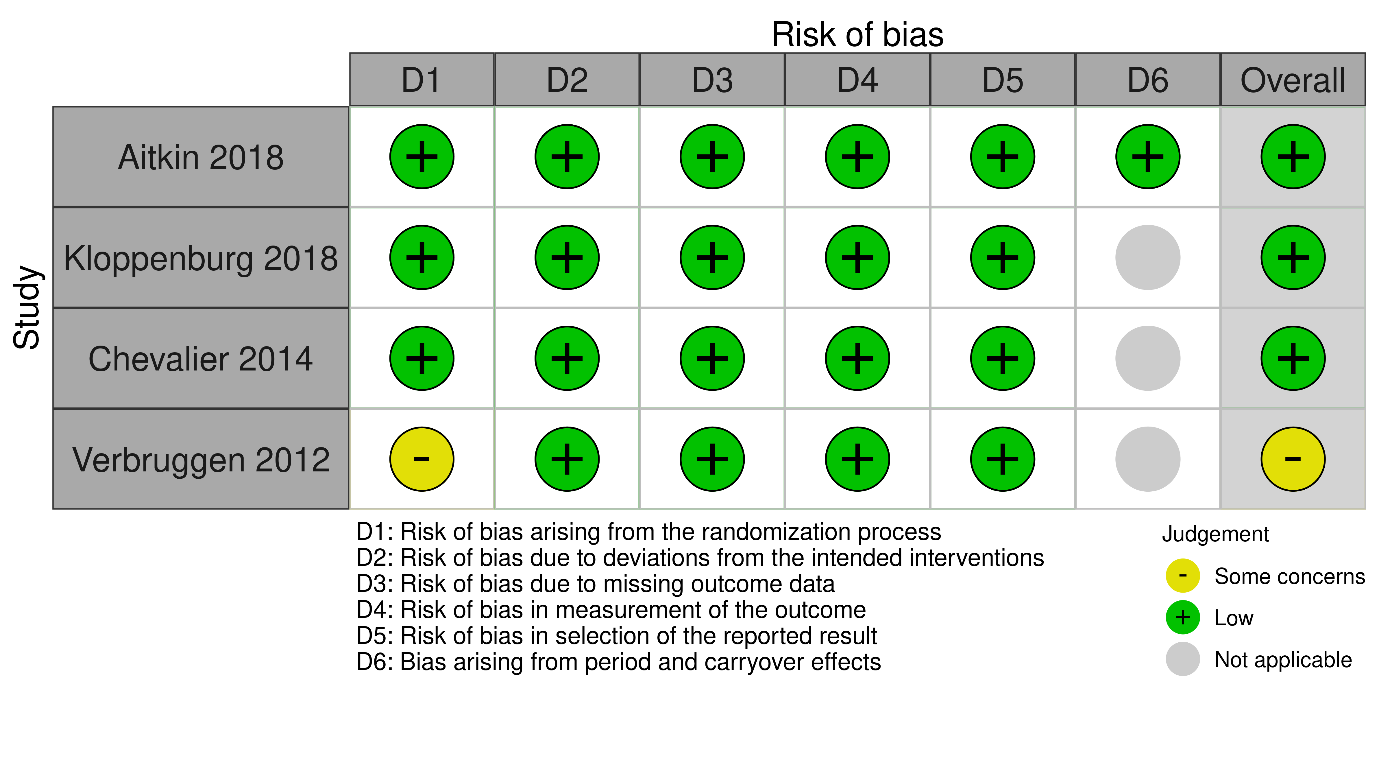

Supplement: Multimedia component 1 [file mmc1.docx]
